# 869. Evaluation of the Impact of Polymicrobial Bloodstream Infections (BSIs) on the Performance of BioFire Blood Culture Identification 2 (BCID2) Multiplex Polymerase Chain Reaction (PCR) Panel

**DOI:** 10.1093/ofid/ofad500.914

**Published:** 2023-11-27

**Authors:** Jonathan S Lapin, Aishwarya Gogineni, Karan Singh, Nina Gao, J Kristie Johnson, Kimberly C Claeys

**Affiliations:** University of Maryland Medical Center, Baltimore, Maryland; University of Maryland School of Pharmacy, Baltimore, Maryland; University of Maryland School of Pharmacy, Baltimore, Maryland; University of Maryland School of Medicine, Baltimore, Maryland; University of Maryland School of Medicine, Baltimore, Maryland; University of Maryland Baltimore, Baltimore, MD

## Abstract

**Background:**

Rapid diagnostic tests (RDTs) have led to a paradigm shift in the management of BSIs, allowing for earlier species identification and decreased time to appropriate antimicrobials. The University of Maryland Medical Center recently switched from Verigene, a nucleic acid microarray panel, to BioFire BCID2, a multiplex PCR panel. Whereas Verigene is known to perform poorly in polymicrobial BSIs, it is unknown how BioFire BCID2 performs and how this impacts downstream antimicrobial therapy decisions.

**Methods:**

We performed a retrospective cohort study of adult patients with polymicrobial blood cultures tested with BioFire BCID2 between 01/01/2022 and 05/31/2022. Family, genus, species, and resistance identification from BioFire BCID2 were compared to conventional microbiological methods and phenotypic antimicrobial susceptibility testing (AST) to assess the on-panel accuracy of BCID2 in polymicrobial BSI. Antimicrobial prescribing based on BioFire BCID2 results was collected to determine how the BCID2 impacts decision making in polymicrobial BSIs.

**Results:**

Among 504 patients with positive blood cultures, 52 (10.3%) were polymicrobial BSIs. Among these 52 BSIs, 91 distinct organisms were identified using BCID2, and 19 *Enterobacterales*, 33 *Staphylococcus* spp., and 14 *Streptococcus* spp. identified at the family or genus level. Additionally, 105 organisms and 19 Coagulase-negative *Staphylococci* were recovered with conventional methods. BCID2 correctly identified 89.4% (101/113) of on-panel organisms at the species level. There was 88.5% concordance between BCID2 resistance identification and phenotypic AST. 36 (69.2%) and 20 (38.5%) patients had antimicrobial regimen changes within 24 hours of BCID2 results and AST, respectively – most were de-escalations (66.7% and 85%, respectively). 12 of these patients had further changes in antimicrobials; 2 of which were escalations due to discrepant BCID2 and AST results.

**Conclusion:**

On-panel agreement between BioFire BCID2 and conventional methods was high in polymicrobial BSI, although less than reported in studies assessing only monomicrobial BSI. In patients with polymicrobial BSIs, the implementation of BCID2 allowed for early changes to antimicrobial therapy.

**Disclosures:**

**Kimberly C. Claeys, PharmD**, Abbvie: Advisor/Consultant|bioMérieux Inc.: Advisor/Consultant|bioMérieux Inc.: Speaker|La Jolla Pharmaceuticals: Advisor/Consultant|Melinta Therapeutics: Advisor/Consultant

